# Past, present and future in low-risk myelodysplastic syndrome

**DOI:** 10.3389/fmed.2022.967900

**Published:** 2022-07-15

**Authors:** Selami Kocak Toprak

**Affiliations:** Department of Hematology, Ankara University School of Medicine, Ankara, Turkey

**Keywords:** myelodysplastic syndrome, low risk, treatment, anemia, thrombocytopenia

## Abstract

Myelodysplastic syndromes (MDS) is a heterogeneous group of disorders characterized by increased risk of acute myeloid leukemia transformation and cytopenia. The prognosis of MDS patients can be evaluated with various scoring systems, the most commonly used are IPSS (International Prognostic Scoring System), revised-IPSS, and WPSS (WHO classification-based prognostic scoring system). MDS treatment is decided according to the risk classification. The goal of treatment in low-risk MDS is to improve cytopenia, reduce transfusion needs, improve quality of life, prolong overall survival, and maybe reduce the risk of progression to leukemia. In the near future, combining both genomics-based, *ex vivo* functional based and molecular stratification analysis will lead the way to a personalized and targeted approach.

## Introduction

Myelodysplastic Syndrome (MDS) can be defined as a common name given to a heterogeneous group of diseases in which ineffective hematopoiesis and cytopenia are predominate. There are specific characteristic changes in blood and bone marrow, mainly dysplasia (dyserythropoiesis, dysgranulopoiesis and monocytosis, dysmegakaryopoiesis) in all three series of bone marrow.

Although it can be observed in almost any age group, MDS, which is known to affect mostly the elderly, poses an important problem for hematologists especially in terms of diagnosis and ability to determine and administer an appropriate treatment in limited time (1). The median age at diagnosis is 71, and the annual incidence of disease is 0.1/100,000 for the population under the age of 40, while this rate is 30.2/100,000 for the 70–79 age group and 59.8/100,000 for those over 80 years old, according to the data of the United States (US) National Cancer Institute (NCI) (2, 3).

Cytopenia, bone marrow dysplasia, and some characteristic chromosomal anomalies define this disease according to the World Health Organization (WHO); however, understanding the pathogenesis, classification, and prognosis of the disease has actually become much easier as a result of the integration of next-generation DNA sequencing (NGS) technique into daily practice and its integration with morphology, cytogenetic and molecular genetic techniques (2).

The highly heterogeneous nature of MDS complicates the treatment of the disease and requires individualization as well. The only “curative” approach among many treatment options actually comes with allogeneic hematopoietic stem cell transplantation (AHSCT), but this form of treatment can unfortunately be applied to a limited number of “fit” patients. For the majority of patients, the preferred treatment methods are generally “non-intensive” options due to age, comorbidities, etc. of these patients, and the ideal choice is to use risk-based approaches/treatments. Almost all of a wide range of treatment options ranging from growth factor to lenalidomide and hypomethylating drugs are the preferred approaches to correct cytopenia, improve quality of life, and if possible, prevent disease progression, rather than radical treatment of the disease.

In this review, the past, present and possible future view of low-risk MDS management will be discussed including general definition, classification, clinical presentation, risk stratification, prognostic evaluation.

## Definition, classification, pathogenesis, clinical presentation, risk stratification and prognostic assessment of myelodysplastic syndrome

### Definition

Myelodysplastic syndrome is a wide spectrum of heterogeneous diseases in which different sizes of cytopenia and morphological dysplasia that have the risk of developing into acute myeloblastic leukemia (AML) (2). It is clear that the most important determinant for MDS is dysplasia, which can be detected in early and mature cells of the bone marrow, rather than the presence of cytopenia.

Dyserythropoiesis is identified by various anomalies in the nucleus and cytoplasm of erythroid cells. In the nucleus, budding, bridging, karyorexia, presence of multiple nuclei and megaloblastoid changes are present; cytoplasmic ones are classified as ring sideroblast, vacuolization and Periodic acid–Schiff (PAS) positivity (2). Dysgranulopoiesis, on the other hand, is characterized by very small or abnormally large myeloid lineage cells, nuclear hyposegmentation (pseudo-Pelger-Huet), nuclear hypersegmentation, reduction or absence in granulation, the presence of Pseudo-Chédiak-Higashi granules, Döhle bodies, Auer rods, and Barr bodies (2). Dysmegakaryopesis is classified as the presence of micromegakaryocytes, nuclear hypolobation, and multiple nuclei (2).

### Classification

It was first named as preleukemia in 1953, and as a result of various definitions such as chronic erythremic myelosis, hypoplastic acute myeloid leukemia, and dysmyelopoietic syndrome, its first morphological classification (FAB, French–American–British) was made in 1982 using the name MDS (2, 4). WHO updated this classification in 2001 and 2008, and finally, in 2016, made a morphological and cytogenetic-based classification and defined 6 disease types in general, including subgroups (5). It should be emphasized, however, that the issue of exactly where MDS and AML diverge continues to be debated. The only difference between the two diseases is not only the number of blasts, but also the clinical progression rate, as well as the biological and morphological characteristics of the diseases. Especially, the US National Comprehensive Cancer Network (NCCN) panel team and also WHO state that a disease with a blast rate of between 20 and 29% and a stable clinical course for at least 2 months can be defined as “high-risk MDS” (3). However, in this situation, it would be more accurate to define patients with FLT3 or NPM1 mutations as AML rather than MDS (2, 6).

### Pathogenesis

Myelodysplastic syndrome is known to be a hematopoietic stem cell disease that develops as a result of the interaction of bone marrow microenvironment and immune system with various genetic and epigenetic factors for many years (2). In MDS, it was reported that structural genetic defects, mostly caused by various “unbalanced changes” due to chromosomal losses and excesses, such as deletion (del) 5q, monosomy 7, trisomy 8, and del 20q and were identified in almost more than 50% of cases (7). With the adaptation of the NGS technique to clinical practice, recurrent mutations of more than 50 genes involved in DNA methylation, chromatin modification, RNA *“splicing”, “cohesion”* formation, transcription control, and DNA repair and signaling processes have been detected (2, 8). It has been suggested that there are various immunological imbalances, especially in the T lymphocyte series; for example, cytotoxic T cells increase in low-risk MDS and regulatory T cells dominate the presentation together with the immune “escape” mechanism in the high-risk subgroup (2, 9). Its shown that low risk MDS patients also have chronic inflammation (10).

Another MDS developmental process for which the underlying mechanism is largely unknown is “therapy related” disease, which is cytotoxic drug or radiotherapy related. The remarkable feature here is that the disease develops at a higher rate in individuals carrying a CHIP (clonal hematopoiesis of indeterminate prognosis) clone (11).

### Clinical presentation

Long-standing macrocytic anemia, mild thrombocytopenia, and neutropenia may be identified during the development of MDS before the disease clinically develops. In fact, the diagnosis can be made by identifying cytopenia in routine blood tests by chance even when the patient is not more symptomatic and at relatively earlier times.

Complaints such as weakness, fatigue, decrease in effort capacity, dizziness, and cognitive dysfunction, which are triggered by tissue hypoxia related to anemia and caused by the fact that almost the whole organism, mainly the musculoskeletal system, cardiovascular system and central nervous system are affected, are remarkable. Although mild at first, skin/mucosal bleeding caused by overt/deep thrombocytopenia is not surprising in cases in whom the diagnosis is delayed. Thrombocytopenia can mistakenly be diagnosed as immune thrombocytopenia if dysplastic changes are ignored and not recognized. Neutropenia accompanied by functional disorders may be the cause of life-threatening bacterial and fungal infections. There is a substantial number of MDS cases diagnosed with fever, cough, dysuria, and even septic shock.

### Risk stratification and prognostic assessment

In the historical process, many approaches have been developed that try to determine the prognostic classification through defining a MDS patient and consider various criteria such as clinical features, bone marrow blast rate, cytopenia, age, lactate dehydrogenase level, and cytogenetic features (2). The first of these was the International Prognostic Scoring System for MDS-IPSS, which divided patients into four subgroups in 1997 according to their cytopenia, bone marrow blast rate and cytogenetic features, and their median survival of 0.4–5.7 years (12). In the last 15 years, the need for a new classification has arisen as a result of the addition of ferritin, beta 2 microglobulin, bone marrow fibrosis, comorbidity, and performance status of patients, morphological re-classification of MDS, and finally the new cytogenetic features that can be detected with the developing technology (2). With the evaluation of 7,012 patients belonging to the database of the MDS study group (International Working Group for Prognosis in MDS-IWG-PM), which was formed with international multicentre participation, a more detailed scoring system -revised (R) IPSS- was developed which defines 5 prognostic categories with five different cytogenetic features, and classifies the depth of cytopenia and bone marrow blast ratio in more detail (13) (Table 1). Moreover, a WHO classification-based prognostic scoring system (WPSS) was developed with the participation of two centers from Italy and Germany in 2007, and five different subgroups were defined with a median survival of 12–103 months (14) (Table 2). Although anemia is a poor prognostic subgroup in this classification, the depth of anemia, in other words Hb level, was also included in the prognostic classification in a recent analysis as its depth and its clinical reflection were not sufficiently correlated (15). IWG-PM reported in its analysis comparing WPSS with R-IPSS that WPSS was also a very effective scoring method in the prognostic classification of untreated MDS cases and AML transformation (16).

**TABLE 1:** R-IPSS scoring system (13).

Prognostic score value							
	**0**	**0.5**	**1**	**1.5**	**2**	**3**	**4**
**Prognostic category**							
Cytogenetics	Very good		Good		Intermediate	Poor	Very poor
BM blasts, %	≤2		>2 to <5		5–10	>10	
Hgb, g/dl	≥10		8 to <10	<8			
Platelets, ×10^9^/L	≥100	50 to <100	<50				
ANC, ×10^9^/L	≥0.8	<0.8					

ANC, absolute neutrophil count; BM, bone marrow; Hb, hemoglobin.

Cytogenetics: Very good: −Y, del(11q); Good: Normal, del(5q), del(12p), del(20q), del(5q) + 1 additional abnormality; Intermediate: del(7q), + 8, + 19, i(17q), other abnormalities not in other groups; Poor: −7, inv(3)/t(3q), −7/del(7q) + 1 additional abnormality, complex (three abnormalities); Very Poor: Complex (>3 abnormalities).

**TABLE 2:** WHO classification-based prognostic scoring system (WPSS) scoring system (14).

Score				
**Parameter**	**0**	**1**	**2**	**3**
WHO category	RA, RARS, 5q-	RCMD, RCMD-RS	RAEB-1	RAEB-2
Karyotype	Good^a^	Intermediate^b^	Poor^c^	–
Transfusion	Yes	Regular	–	–
**Score**		**Risk subgroup**	**Survival, Italian cohort (m)^d^**	**Survival, German cohort (m)^d^**
0		Very low	103	141
1		Low	72	66
2		Intermediate	40	48
3–4		High	21	26
5–6		Very high	12	9

^a^Good: normal, −Y, del(5q), del(20q).

^b^Intermediate: other abnormalities not seen in “good” or “poor”.

^c^Poor: complex (≥3 abnormalities) or chromosome 7 anormalies.

^d^Median survival.

Although there are many different approaches today, it is clear that the R-IPSS risk classification system is generally used in making treatment decisions by considering the age and performance status of a patient (2, 3).

The presence of various somatic mutations which are not in these systems, but have been identified with NGS in recent years, such as TP53, ASXL1, EZH2, ETV6, and RUNX1 that lead to poor clinical course, and SF3B1 that leads to a good clinical outcome, are tried to be integrated into scoring systems by some centers (17–19).

## Treatment in low-risk myelodysplastic syndrome

International prognostic scoring systems that divide MDS into two large groups as low or high risk have actually revealed two disease subtypes with completely different treatment goals (20). While treatment policies in low-risk patients focus mostly on the correction of cytopenia which is reducing blood product support, especially erythrocyte suspension support, improving the quality of life, and maintaining it, if possible; those in the high-risk subgroup are correction of cytopenia, as well as delaying leukemic progression, and if possible, ensuring the survival of patients (Table 3) (21).

**TABLE 3:** Treatment goals in low- and high-risk myelodysplastic syndromes (MDS) patients (20).

Order of priority	MDS risk classification	
	Low-risk	High-risk
1	Management of cytopenia Fewer transfusions Less iron load	Delaying disease progression Prolonging survival Recovery
2	Sustainability of the administered treatment Improving and maintaining quality of life	Reducing the load of disease Management of cytopenia Fewer transfusions
3	Delaying disease progression Prolonging survival	Sustainability of the administered treatment
4	Recovery	Improving and maintaining quality of life

There is no conflict regarding the inclusion to low-risk MDS of patients who belong to “low” and “intermediate-1” risk groups according to the IPSS, and “very low” and “low” risk groups according to the R-IPSS. However, there is a conflict as to whether patients in “intermediate” risk group in R-IPSS have low or high risk. While some researchers place those in the “intermediate” risk group directly into the high-risk MDS group, others consider those in the “intermediate” risk group above 3.5 points only as high-risk MDS (20, 21). Nevertheless, it is obvious that the first things to consider when making a decision are the worsening of disease or side effects might be caused with the treatment.

### Watch and wait/observation

In fact, there is no need to move beyond “supportive” therapy due to the presence of mild and asymptomatic cytopenia in a substantial number of low-risk MDS patients. This approach can be considered applicable to all low- or high-risk MDS patients who do not have a long-life expectancy due to age and comorbidities. Patients who are fit, asymptomatic, without blast increase, and who do not have a high-risk cytogenetic/molecular profile do not require any special treatment other than regular controls (21, 22). Results from a recent real-world cohort study (*n* = 125) indicate that over a third of patients with low-MDS have been managed using watchful waiting only, with no systemic treatment or transfusions received (23). However, it is recommended that asymptomatic low-risk MDS patients should be followed up more closely if they have various mutations that are not currently included in the risk classification and show a genotypically high-risk prognostic profile, such as ASXL1 mutation (21). The most important markers in these patients are rapid worsening cytopenia, an increase in the number of blasts in the peripheral blood or bone marrow, and the change of findings in cytogenetic/molecular studies (21).

### Treatment options in symptomatic patients

#### Treatment of anemia

Current treatment approach in low-risk MDS patients focuses on combating cytopenia, especially anemia, and the poor results of transfusion load.

##### Treatment with erythropoiesis stimulating agents

Symptoms reflecting anemia-related tissue hypoxia such as weakness, fatigue, and decreased exercise capacity are the most frequently reported complaints in MDS patients. These patients also need regular erythrocyte suspension (ES) support. Regular and frequent ES support, on the other hand, means a very problematic complication such as transfusional hemosiderosis, as well as a financial load and the need for social support.

The use of “erythropoiesis-stimulating agents” such as recombinant erythropoietin (EPO) or darbepoetin (DAR) as a single drug has been the mainstay of treatment for many years in low-risk patients who are at the forefront of anemia and need frequent transfusion support. On the other hand, phase three randomized studies on both drugs were conducted more recently and the use of EPO-alpha was eventually approved (20, 21). In a multicenter, randomized phase 3 study from European countries, 147 low-risk MDS patients with hemoglobin (Hb) level of ≤10 g/dL, EPO level of ≤500 mU/mL, and low transfusion load were randomized to receive DAR-alpha or placebo at the rate of 2/1 (24). Following placebo or subcutaneous administration of 500 μg DAR-alpha every 3 weeks for 24 weeks, the frequency of transfusion was significantly higher in the placebo arm (59.2 vs 36.1% and *p* = 0.008), while the recovery of anemia was significantly higher in the DAR arm (14.7 vs 0% and *p* = 0.016). In another multicenter, double-blind, placebo-controlled phase 3 study from European countries, 130 low-risk (IPSS low and medium-1) MDS patients with similar characteristics to the other study were randomized to EPO-alpha or placebo arms at the rate of 2/1 (25). After subcutaneous administration of 450 IU/kg/w EPO-alpha or placebo for 24 weeks, the recovery of anemia was significantly higher in the EPO arm (31.8 vs. 4.4% and *p* = 0.001).

Some features in low-risk MDS patients have become important in predicting the response to “erythropoiesis stimulating agents.” These features are low endogenous EPO level (<500, <100 IU/L) and having a total ES transfusion load of less than 4 units in 2 months (26, 27).

In European Union countries, endogenous EPO level should be <200 IU/L for reimbursement approval of EPO-alpha, whereas in our country this value is determined as <500 IU/L. In many low-risk MDS patients, the effect of “erythropoiesis stimulating agents” becomes apparent in about 3 weeks, while it is also stated that this effect can be sustained for a median of 15–18 months (21).

In patients whose expected response cannot be obtained with “erythropoiesis stimulating agents” and especially in the subgroup with ring sideroblasts, recovery can be achieved in approximately 20% of patients with subcutaneous application of granulocyte colony stimulating factors (G-CSF) of 1–2 μg/kg/w (28).

##### Lenalidomide

In del(5q) positive low-risk MDS patients with low transfusion load and symptomatic anemia, the first-line treatment of anemia is again “erythropoiesis stimulating agents” (26). However, it is noteworthy that most of these patients have high endogenous EPO levels, usually associated with low and short-term response rates and correlated with high clonality rate in myeloid precursor cells (23). Lenalidomide seems to be a good choice with a treatment success of up to 70% in the treatment of anemia, especially for patients with high EPO levels and a constant need for ES transfusion (29, 30). The most common side effects of the drug are diarrhea, rash, nausea, constipation, fever, itching, shortness of breath, recurrent arterial thrombotic events, and hematological side effects such as neutropenia and/or thrombocytopenia, and it is obvious that these complications should be considered very important considering the average age and fitness of MDS patients (20).

In an international multicenter, randomized, phase 3 study published in 2016, 239 low-risk and del(5q) negative patients were randomized 2/1 to lenalidomide or placebo arm (31). ES transfusion independence was clearly superior in the lenalidomide arm, while no significant deterioration in quality of life was detected in the drug arm. In another recent randomized study, 131 patients resistant to “erythropoiesis stimulating agents”, who were del(5q) negative low-risk and ES transfusion-dependent, were randomized to either lenalidomide alone or lenalidomide + EPO arms (32). The use of lenalidomide in combination with “erythropoiesis stimulating agents” provided a significant superiority in anemia response compared to the arm in which it was given alone (39.4 vs 23.1% and *p* = 0.044). In a recent study, the combination of lenalidomide and erythropoiesis stimulating agents yields 38.9% of major erythroid responses who relapsed or unresponsive to erythropoiesis stimulating agents (33).

On the other hand, patients with TP53 mutations, which constitute approximately 20% of all patients, did not have the expected benefit from lenalidomide with high leukemic transformation rates (21). In the LEMON5 study, the overall response rates and survival in patients with TP53 mutation who were followed up with lenalidomide monotherapy were significantly lower than those in patients without TP53 mutation (34). Moreover, TET2 and RUNX1 mutations are associated with poor outcome and lenalidomide unresponsiveness (21). Even if there is no disease progression or leukemic transformation in patients who do not respond adequately to lenalidomide in the presence of TP53 or other mutations, it is recommended to administer hypomethylating (HMI) agents or HSCT in the absence of an ongoing clinical trial (21).

##### Supportive therapy with iron chelation

Many MDS patients live dependent on regular ES transfusions as part of supportive therapy. In these patients, “non-transferrin bound iron” and labile plasma iron fraction increase, and ultimately and stored especially in the heart, liver and endocrine glands. In a phase 3 study comparing placebo and deferasirox, there was a significant risk reduction of 36.4% in event-free survival (event: worsening cardiac function, hospitalization with heart failure, liver dysfunction, liver cirrhosis, and AML transformation) in the iron chelation group (35).

Many international guidelines recommend iron chelation based on the ferritin level; the opinion is that this amount should be at least 1,000 ng/mL (21). It is known that the most commonly used chelator is deferasirox, and the patient compliance problem is also improved with the film-coated tablets released in recent years. Iron chelation is an application that must be included in the algorithm before HSCT, and it positively affects the transplant outcome.

##### Immunosuppressive drugs

Immune dysregulation is better understood in the etiopathogenesis of MDS and is considered to be responsible for ineffective hematopoiesis. In this context, it was reported that very satisfactory response rates (16–67%), including all three series, were obtained with antithymocyte globulin (ATG) and/or cyclosporine treatment (31). Some features become important in predicting patients who will benefit from immunosuppressive therapy. Presence of dysplasia which is the subtype previously classified as refractory anemia, absence of ring sideroblast subtype, especially hypoplastic/hypocellular bone marrow, having HLA DR15 typing, young age (<60 years), female gender, presence of trisomy 8, and relatively short duration of transfusion need are among these characteristics (21). Interestingly, in a retrospective study involving a large number of patients, in which the presence of SF3B1, a somatic mutation known to be associated with good clinical outcome in MDS, adversely affected the overall response, it was reported that a total response rate of up to 45% was obtained with the use of horse-derived ATG (32). A meta-analysis was unable to associate specific biomarkers predictive of response given the overall lack of prospective, randomized controlled studies for the use of immunosuppressive treatment in low risk MDS (33).

##### Hypomethylating agents

Hypomethylating agents (HMIs), used at standard or reduced doses, are included in the treatment of low-risk MDS. Although it is generally approved by the US Food and Drug Administration (FDA), it has a more limited use in European countries. The reason may be disappointing results obtained in the studies. In a phase 2 study of the Nordic group, which included patients with low-risk MDS who were resistant to the EPO + G-CSF combination or were not suitable for transfusion, a response of only 20% was achieved with azacitidine (AZA) at a dose of 75 mg/m^2^/day administered for 5 days every 28 days. Moreover, this response was both short-term and more toxic than expected (36). Similarly, in prospective studies of the French group, in which they randomized 98 low-risk patients with a median age of 72 years to AZA vs AZA + EPO treatment arms, transfusion independence at the end of six courses was only achieved in 16.3% of patients (14.3% in the AZA-EPO arm) (37). In a study which 113 low-risk MDS patients were included, a total response rate of 70% was achieved with 3 days of 20 mg/m^2^/day decitabine (DEC) every 28 days, while transfusion independence was achieved in 32% of patients (38). Although the oral formulation of AZA, which has been developed in recent years, promises ease of administration and longer lasting efficacy at lower doses, the international multicenter phase 3 study which compared CC-486 (oral AZA) with placebo and included low-risk MDS patients with ES transfusion dependent and thrombocytopenia was terminated earlier than expected due to toxicity (21, 39).

In 2020, the FDA and Canadian authorities approved DEC/cedazuridine (ASTX727 or DEC-C, oral decitabine) for the treatment of all subtypes of adult MDS and CMML in any stage of disease based on the 60% of overall response rate (40). In the Ascertain trial, the low grade MDS patients had 57% overall response rate, 48% became ES independent and 67% platelet transfusion independent (41).

##### New drugs developed for the treatment of anemia

“Luspatercept” and “sotatercept”, also named as “erythropoiesis maturation agents”, are specific activin receptor fusion proteins that act as ligand traps to neutralize negative regulators of late-stage erythropoiesis (2, 20, 21). In the PACE-MDS study which included 57 MDS patients with low transfusion load and in IPSS low and intermediate-1 risk groups, hematological recovery and transfusion independence were reported as 63 and 38%, respectively, in the group using relatively higher dose (0.75–1.75 mg/kg) luspatercept (42). In an international multicenter, double-blind, placebo-controlled, randomized, phase 3 study (MEDALIST) published in January 2020, 229 MDS patients with ring sideroblasts who were in very low, low, and intermediate risk groups according to R-IPSS were randomized 2/1 to luspatercept and placebo arms (43). Transfusion independence (38 vs 13%) and hematological recovery (58 vs 17%) were significantly superior to placebo in the luspatercept arm, while AML transformation in both arms was not different. In April 2020, the FDA approved luspatercept for use in MDS patients with ring sideroblasts who are with very low, low, and intermediate risk and who require two units or more of ES for 8 weeks and have not benefited from “erythropoiesis stimulating agents”.

Roxadustat, on the other hand, is a small molecule that can be used orally and is an inhibitor of the “hypoxia-inducible factor” “prolyl hydroxylase” (20). There are ongoing studies on the use of this drug, which stimulates endogenous EPO production, eliminates the negative effects of inflammation in endogenous EPO production, triggers erythropoiesis and Hb production, and regulates iron regulation through hepcidin metabolism, in anemia in MDS and chronic kidney disease (21). The data for the low burden transfusion dependent low risk MDS are promising which 38% of the patient achieved transfusion independency over 8 weeks in weeks 1–28 of treatment while 42% achieved this during 52 weeks of treatment (44). The results of a recent phase 3, randomized, double-blind, placebo-controlled study showed that transfusion independence was achieved in nine patients (37.5%) at 28 and 52 weeks; seven of the patients patients received 2.5 mg/kg dose (NCT03263091) (45).

Imetelstat is a drug that help maintaining normal hematopoiesis by acting as a telomerase inhibitor in cells with short telomere length and hyper telomerase activity (21). Short telomeres and high telomerase activity are associated with shorter overall survival in MDS (2, 20). In a study published at the annual meeting of the American Society of Hematology in 2017, transfusion independence was found in 34% and erythroid-hematological improvement in 63% of patients with imetelstat in MDS patients with low and intermediate-1 risk who were resistant/unresponsive to “erythropoiesis stimulating agents” (46). When administered intravenously every 4 weeks, transfusion independence was reported in 42% of patients and response duration may be over 1 year in 30% of patients up to 2.8 years (47). Furthermore, specific inhibitors of IDH1 or IDH2 genes, ivosidenib and enasidenib showed promising responses (50%) in patients that carry the somatic mutations (48, 49). KER-050, a modified activin receptor type IIA inhibitor, is designed to target transforming growth factor-β ligands, including activin A. In an open label phase II study in very low to intermediate risk MDS patients, overall erythroid response rate was 60% (*n* = 6/10). 33% (*n* = 1/3) non-transfused participants had a hemoglobin increase of ≥1.5 g/dL sustained ≥8 weeks. Increases in platelets were also observed (50). The new treatment approaches are given in Table 4.

**TABLE 4:** New treatment strategies in low grade myelodysplastic syndromes (MDS).

Drug	Mechanism of action
Luspatercept and sotatercept	Activin receptor fusion proteins that act as ligand traps to neutralize negative regulators of late-stage erythropoiesis
Roxadustat	Inhibitor of the “hypoxia-inducible factor” “prolyl hydroxylase”
Imetelstat	Telomerase inhibitor in cells with short telomere length and hyper telomerase activity
Ivosidenib and Enasidenib	Specific inhibitors of IDH1 or IDH2 genes

#### Treatment of thrombocytopenia

In low-risk MDS with thrombocytopenia, platelet suspension transfusion and thrombopoietin (TPO) receptor agonists, in addition to HMIs, constitute a remarkable treatment option (21). In a randomized study in which “romiplostim” was compared with placebo in low-risk MDS patients, it was found that the platelet counts increased, bleeding episodes and the need for thrombocyte suspensions decreased significantly in patients who received romiplostim compared to the placebo group (51). In the 5-year long-term analysis of the same study, transformation to AML and death rates were not different in the drug group than in the placebo group (52). In a prospective, multicenter EUROPE phase II trial, mutated SRSF2 occurred more often in responders of romiplostim compared with non-responders (41 vs 16%, *p* = 0.018) (53).

**FIGURE 1:**
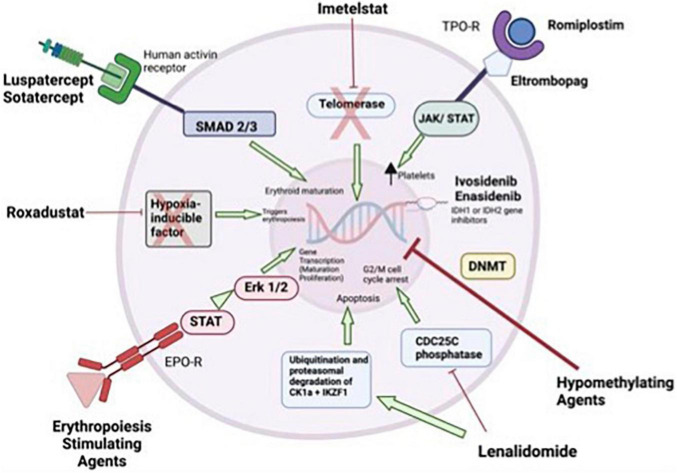
Mechanism of actions for treatments in low-risk myelodysplastic syndromes (MDS). Erythropoiesis stimulating agents stimulate gene transcription of maturation and proliferation of erythrocytes through JAK/STAT and Erk1/2. Lenalidomide inhibits the CDC25C phosphatase and by decrease in CK1α levels. Inhibition of the CDC25C phosphatase leads to a stoppage of proliferation by induction of an arrest in the cell cycle at the transition between G2 and M phase. Hypomethylating agents induce DNA hypomethylation. Ivosidenib, and enasidenib inhibits IDH1 or 1DH2 genes. Thrombopoietic receptor agonists activate signaling leads to increased platelet production. Imetelstat is a drug that help maintaining normal hematopoiesis by acting as a telomerase inhibitor in cells with short telomere length and hyper telomerase activity. “Luspatercept” and “sotatercept” specific activin receptor fusion proteins that act as ligand traps to neutralize negative regulators of late-stage erythropoiesis. Roxadustat is a small molecule that can be used orally and is an inhibitor of the “hypoxia-inducible factor” (20, 21, 61–64). EPO-R, erythropoietin receptor; JAK, janus kinase; STAT, signal transducer and activator of transcription; TPO-R, thrombopoietin receptor.

In a phase 2 study in which 90 low-risk thrombocytopenic MDS patients were randomized to “eltrombopag” and placebo at a rate of 2/1, decrease in thrombocyte response and bleeding episodes were found to be significantly superior in the eltrombopag arm (47 vs. 3%, *p* = 0.0017 and 14 vs 42%, *p* = 0.0025, respectively) (54). AML transformation was also reported to be same between the two groups. A similar response rate of 44% was observed in a second phase 2 dose escalation study. The predictors of response were the presence of a PNH clone, marrow hypocellularity, thrombocytopenia, and baseline elevated plasma TPO levels (55). The combination of eltrombopag and lenalidomide in low and intermediate risk MDS demonstrated good efficacy with ORR of 40.9%, response durability and an acceptable safety profile (56). The mechanisms of potential treatment alternatives for low grade MDS are summarized in Figure 1.

#### Allogeneic hematopoietic stem cell transplantation

It is clear that performing AHSCT earlier in the course of the disease will provide a more favorable outcome in the long term. On the other hand, it is necessary to avoid a treatment process that carries a substantial risk of death in low-risk patients who have a high chance of obtaining a response with standard first-line “soft” treatment options (57). General opinion is that IPSS low and moderate-1 risk subgroup patients do not have any indications for AHSCT at the time of diagnosis, except IPSS intermediate-1 risk subgroup with cytopenia and/or poor cytogenetic karyotype (58). In a multicenter biologic assignment trial, reduced intensity AHSCT showed advantage to hypomethylating therapy or best supportive care on 3-year OS (47.9 vs 26.6%, *P* = 0.0001) and 3-year leukemia-free survival (35.8 vs 20.6%, *P* = 0.003) in subjects 50–75 years of age with intermediate-2 or high risk MDS (59). In the retrospective analysis of the European Society for Blood and Marrow Transplantation (EBMT), which included 246 MDS patients with low and moderate-1 risk according to IPSS, a 3-year overall survival rate of 58% was achieved with AHSCT and the non-relapse mortality rate was reported to be 30% (60). Therefore, in patients with unresponsive disease to first-line therapies and having poor prognostic characteristics such as life-threatening infection, grade 2 or greater bone marrow fibrosis, severe thrombocytopenia, severe neutropenia, severe anemia, ES transfusion dependency, high-risk molecular anomalies, and treatment-related MDS, AHSCT should be considered an option as an individualized treatment approach (21, 57).

## Conclusion and summary of recommendations for low-risk myelodysplastic syndrome treatment

In conclusion, for the majority of MDS patients, the therapeutic approach is based on IPSS (or IPSS-R/WPSS) stratification, with some non-curative options, except AHSCT. However, it should be known that the heterogeneity and complexity of low risk MDS requires a personalized management that, unfortunately, does not yet exist (20).

**FIGURE 2:**
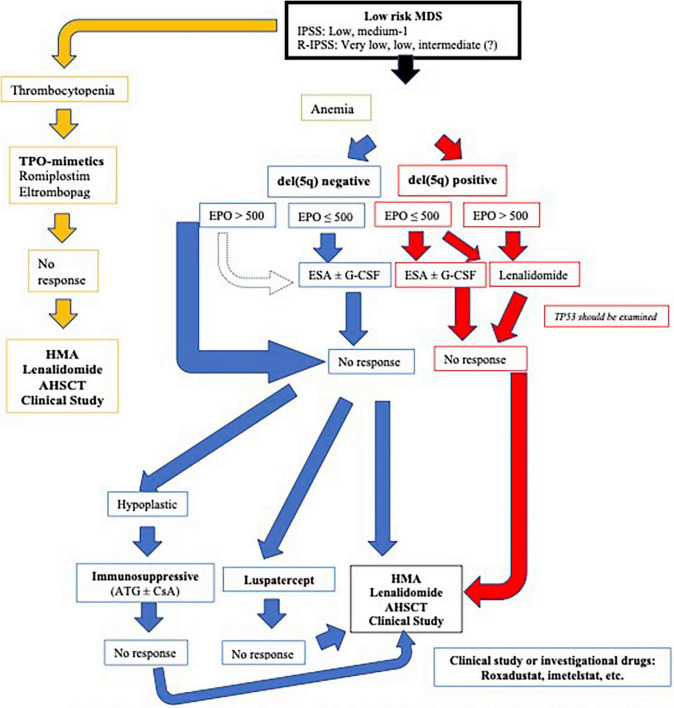
Current treatment approaches in low-risk myelodysplastic syndromes (MDS) (19, 20). MDS, myclodysplastic syndrome*;* IPSS, International Prognostic Scoring System; R-IPSS, Revised International Prognostic Scoring System; TPO, thrombopoietin; EPO, erythropoietin; ESA, erythropoiesis stimulating agents; G-CSF, granulocyte colony stimulating factor; HMA, hypomethylating agents; AHSCT, allogeneic hematopoietic stem cell transplantation; ATG, antithymocyte globulin; CsA, cyclosporine.

It should be kept in mind that there are a substantial number of asymptomatic patients who can only be kept under close follow-up with the option of “watchful waiting”. It is known that “erythropoiesis stimulating agents” in low-risk MDS where anemia is at the forefront and lenalidomide in those with del (5q) positive are beneficial. The combination of lenalidomide + G-CSF may be a good alternative for patients who are del (5q) negative and are resistant to “erythropoiesis stimulating agents”. Immunosuppressive treatment options should not be disregarded, especially in subtypes with low ring sideroblasts, high endogenous EPO levels and additionally HLA DR15 positivity. Although thrombocytopenia and neutropenia are encountered less frequently, they are two characteristics that are more difficult to treat (21). HMIs can be used at adjusted doses and with profit/loss calculations in low-risk MDS patients with both anemia and thrombocytopenia/neutropenia, those who do not respond to the first-line treatments that are specified, and those who have unfavorable somatic mutations. Although mortality rates are high, in patients who are resistant to first-line therapy, are transfusion-dependent, and have additionally high-risk molecular anomalies, the option of AHSCT should be considered without delay, by explaining the possible complications to the patient and family at the appropriate time and in the appropriate order. Recommendations are summarized in Figure 2 (20, 21).

It seems impossible to consider a single gold standard treatment option that will be successful in a large proportion of patients because of underlying stem cell disease and the combination of many different mechanisms in its etiopathogenesis (2). However, with the analysis of the results of ongoing *ex vivo* functional studies and genomic-based studies, it may be possible in the non-distant future to create more specific treatment options that will work in low risk MDS.

## Author contributions

ST conceived and designed the review, collected the data, wrote the manuscript, and approved the submitted version.

## Conflict of Interest

The author declares that the research was conducted in the absence of any commercial or financial relationships that could be construed as a potential conflict of interest.

## Publisher’s Note

All claims expressed in this article are solely those of the authors and do not necessarily represent those of their affiliated organizations, or those of the publisher, the editors and the reviewers. Any product that may be evaluated in this article, or claim that may be made by its manufacturer, is not guaranteed or endorsed by the publisher.
